# Mental health coping strategies and support needs among marginalised further and higher education students in the UK: A cross-sectional study

**DOI:** 10.1371/journal.pmen.0000046

**Published:** 2024-06-17

**Authors:** Shaun Liverpool, Mohammed Moinuddin, Katie Bracegirdle, Jade Eddison, Seyi Joseph, Supritha Aithal, Eve Allen, Parise Carmichael-Murphy, John Marsden, Hayley McKenzie, Ciaran Murphy, Michael Owen, Tasneem Patel, Naseem Akorede Raji, Lynsey Roocroft, Ken Fletcher, Vicky Karkou

**Affiliations:** 1 Department of Social Work and Wellbeing, Edge Hill University, Ormskirk, United Kingdom; 2 School of Medicine and Dentistry, University of Central Lancashire, Preston, United Kingdom; 3 Manchester Institute of Education, The University of Manchester, Manchester, United Kingdom; 4 Lancaster Medical School, Lancaster University, Lancaster, United Kingdom; Xi'an Jiaotong-Liverpool University, CHINA

## Abstract

Students who are marginalised based on varying identities, backgrounds and characteristics are highly vulnerable to mental health challenges, but many do not receive appropriate support from healthcare services. Several barriers have been identified, including cultural and systemic factors. Therefore, everyday coping strategies and support in different settings are vital. This study examines the mental health coping strategies and support needs among marginalised students in the United Kingdom (UK). We analysed qualitative and quantitative data from a cross-sectional survey conducted between December 2021 and July 2022. Statistical analysis was conducted on data obtained using the abbreviated version of the Coping Orientation to Problems Experienced Inventory (Brief-COPE). Qualitative content analysis was applied to data collected using open-ended questions. From a subsample of 788 further and higher education students, 581 (73.7%) students (M = 25 years, SD = 8.19) were categorised as marginalised based on ethnicity, sex/gender, sexuality, religious beliefs, first language, birth country, age (i.e., mature students), and having special education needs/disabilities. Marginalised students had significantly higher scores for problem-focused, emotion-focused and avoidant coping strategies/practices compared to other students. Coping strategies included talking to friends and family, practising religion or spirituality, engaging in creative/innovative activities like hobbies, using entertainment as a distraction, waiting to see if things improve and isolating. Students expressed a need for improved or tailored services, additional academic support, and appropriate social support. These included contemporary approaches to support mental health, such as online provisions, regular mentor/personal tutor meetings, lowered academic pressures and opportunities for organised peer support. The findings from this study highlight significant and timely evidence on coping strategies and support needs among a wide range of marginalised student groups in the UK. This study provides important knowledge that is useful to inform personalised culturally appropriate mental health support that can be offered in education settings.

## Introduction

### Student mental health and wellbeing

The mental health and wellbeing of further and higher education students is a growing public health concern in the United Kingdom (UK) [1–4]. Studies have shown that most mental health disorders develop by the age of 25 years [5], placing students in an at-risk group to experience mental health problems and their related impact. Academic, financial, and social stressors are common factors impacting students’ mental health while at college and university [6–9]. In addition, some students experience role conflicts while juggling employment and caring responsibilities alongside education [10, 11]. With the increased demand for mental health services, UK universities are seeking to expand services and interventions in this area [12]. Therefore, more information is needed to understand the needs of different groups of students to ensure all students can benefit from the services offered. This is especially important for students who are typically disadvantaged or excluded based on sociodemographic characteristics like race/ethnicity, sex/gender and sexuality, from here on, referred to as marginalised students.

Students who are marginalised not only face the general stressors of student life [13, 14], but they are also susceptible to marginal-specific factors. Typically, these factors can increase vulnerability to stressful events in education settings. Such factors include first-generation unfamiliarity with the UK education system [15] and other cultural and language barriers [16, 17]. These instances can result in students having difficulty making friends and developing negative self-identities [10, 11, 14, 18], thereby provoking issues with belongingness, social isolation and feelings of difference [19].

Other aggravating factors that contribute to vulnerability among marginalised students include developing feelings of being unqualified to be at college or university due to receiving overt discrimination, microaggressions and stereotyping [14, 20]. Accordingly, marginalised students are seen as more susceptible to mental health issues, such as depression, anxiety and somatic problems [10, 21–23]. However, the literature suggests that there are several barriers to these students seeking professional support for their mental health, including racism, inequality, stigma, waiting times and lack of knowledge of how to get support [15, 24].

### Accessing mental health support

Students who are marginalised often report barriers to accessing professional mental health services in healthcare settings. Although the incidence of diagnoses in marginalised groups can be high, access to treatment may not always guarantee that a service user’s needs will be met [25]. In recent years, there has been great emphasis on identifying and addressing cultural factors such as competence, attitudes, beliefs, and perceptions among service users and providers of mental health services [26]. However, cultural adaptation of a service, therapy or intervention alone may not ameliorate structural disadvantage. For marginalised students, navigating structural disadvantage in both education and mental health services makes access to health and education-related outcomes difficult [27]. Yet, there is often a focus on outlining how mental health services fail to meet the needs of those who access them, but less is known about what the unmet needs are.

Structural competency has been highlighted as an important facet of global health to address how societal and social injustices shape and sustain health inequities. This framework has been used for understanding how larger social and political structures shape health outcomes and influence the delivery of healthcare services [28, 29]. Therefore, it is important to consider health interventions at the structural level to ensure that the systems of disparity and inequality are targeted [30]. In this context, a better focus on the nature of existing mental health services in education settings, such as the types of mental health interventions offered, who delivers them, and how, can offer insight into what structures sustain inequality in mental health.

### Mental health coping strategies and practices

In the absence of appropriate support, students engage in a variety of coping mechanisms which are sometimes shaped by sociocultural characteristics like gender, age, prior experiences, geographical location, and social groups. Previous research highlighted that problem-solving and social support were the most widely employed coping mechanisms [31]. More specifically, some students commonly use positive coping strategies such as meditation, mindfulness, and physical activities, while other students turn to acceptance, planning, and seeking out emotional support as coping mechanisms [32]. In contrast, dysfunctional coping strategies among some college students include mental and behavioural disengagements, such as avoidance or procrastination, substance use, and social withdrawal or isolation [33, 34]. Among marginalised groups seeking support within their communities, relying on cultural networks and religious institutions for emotional and social support appears to be common [35]. These groups also employ coping strategies that involve processing stressful events on their own and talking about these events to close friends and family [36].

For students from marginalised groups, the importance of an inclusive education environment with responsive support systems is key [37, 38]. In colleges and universities, it is increasingly understood that students benefit from a compassionate environment in which their mental health needs are recognised and where they feel a sense of belonging, supported by empathically attuned university staff [39]. The theoretical underpinnings of this approach can be found in the humanistic psychological frameworks of Maslow [40] and Rogers [41]. Maslow argued that individuals are motivated by two drivers: the need for safety and the need for development, with the need for safety taking precedence [40]. This means that when students feel unsettled and anxious in the classroom, they are more likely to focus on managing this anxious state. Once emotional safety has been established, however, then learning can be accelerated, and students shift their attention outwards and more freely engage with the learning environment. Hence the need for psychological safety is particularly true for students from marginalised groups. This then implies a need for support that is tailored to meet the needs of all students, so they can access personalised culturally appropriate mental health care.

### Aims and objectives of this study

Based on the above, we analysed both qualitative and quantitative data collected from marginalised further and higher education students in the UK to explore opportunities for new intervention development and improved service provision. The specific objectives for the quantitative analysis were to assess the types and prevalence of different coping strategies/practices adopted by marginalised students. The qualitative data analysis was conducted to gain further insights into the different coping strategies/practices and to explore the mental health support needs of marginalised students. Therefore, we sought to answer the following research questions:

1. What strategies/practices are used by marginalised students to cope with stress?
2. What kinds of support do students from marginalised groups need or want in educational settings to support their mental health and wellbeing?

## Methods

### Study design

We adopted a pragmatic epistemological position, underpinned by a convergent mixed methods approach, to help understand the mental health coping and support needs among marginalised further and higher education students in the UK [42]. This study was guided by the standards for conducting and reporting mixed methods research [43, 44] and informed by The Strengthening the Reporting of Observational Studies in Epidemiology (STROBE) recommendations [45].

### Data source and preparation

We conducted an international survey between December 2021 and July 2022 that aimed to examine the mental health and wellbeing of further and higher education students returning to face-to-face (in-person) learning after the COVID-19 pandemic restrictions were lifted [46]. Owing to the aims of the current study, a sample of that dataset was deemed appropriate, since several studies highlighted that the COVID-19 pandemic was a stressful period for students [46–49]. The total sample from the original dataset consisted of N = 1160 post-secondary students recruited internationally from a variety of education settings, including colleges and universities [46].

**Fig 1:**
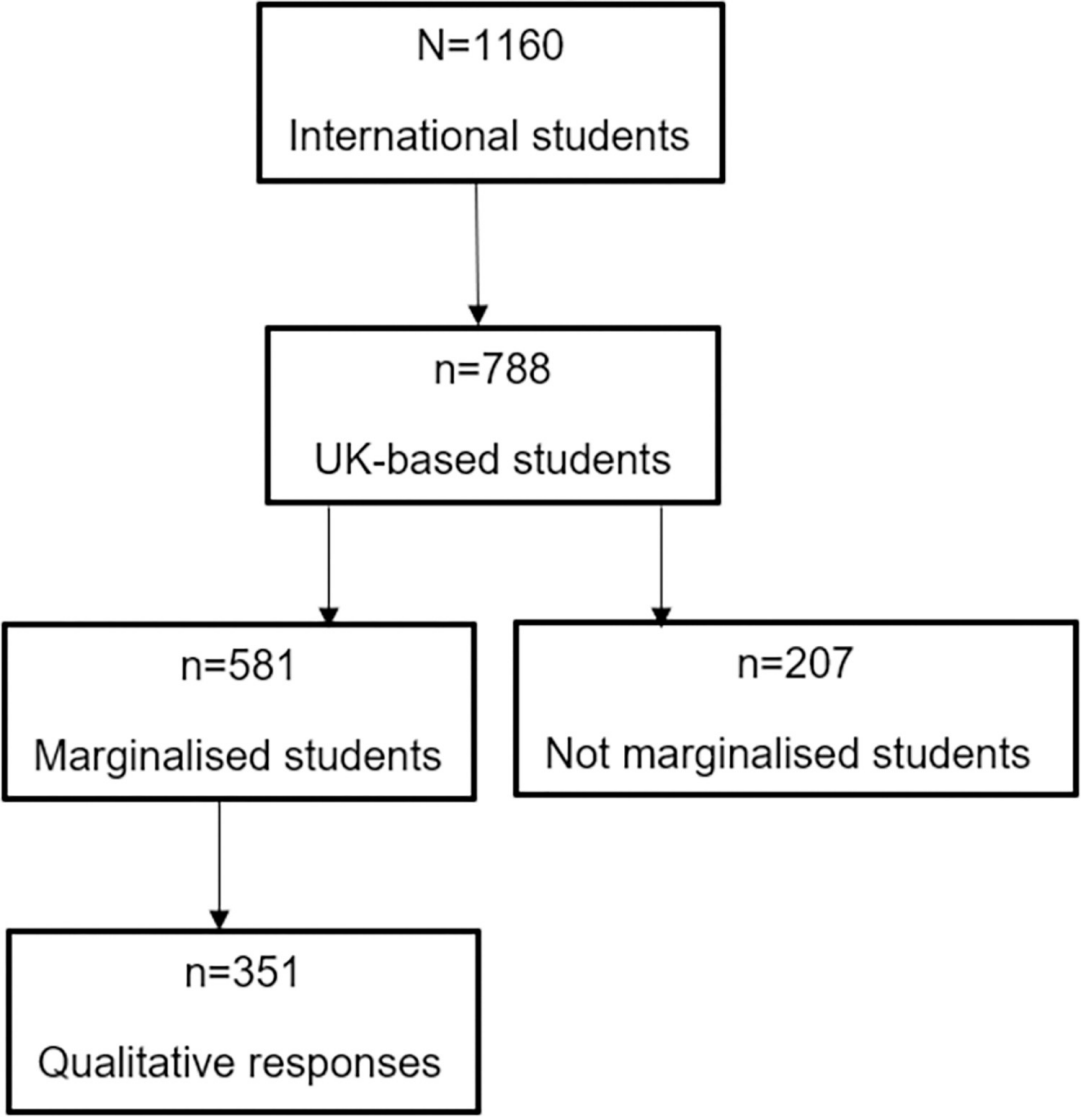
Study sample selection process.

Data were included in the current analysis if the survey respondents were UK-based students (Fig 1). The second inclusion criterion was the completion of any of the key demographic questions that represented age, ethnicity, sex/gender/sexuality, birth country, first language and having special education needs/disabilities. Based on the responses to the demographic questions, students were categorised as marginalised based on the United Nations’ definitions of any group of persons which constitutes less than half of the general population and whose members share common characteristics like ethnicity, sex/gender, sexuality, religion or language, or a combination of any of these [50, 51]. Based on consultations with students, being a mature student and having special education needs were also two important groups that are marginalised in education settings. Further details of the marginalised categories are defined below.

### Data collection questions

#### Demographics

Data from key demographic variables were extracted. Age was measured on a continuous scale using the participant’s date of birth. Ethnicity was captured using self-identified categories (White, Black, Asian, Mixed or Other). Sex and Gender expressions were captured using pronouns (He/Him, She/Her, They/Them or Other) and responding to the question, “Do you identify with the same sex you were assigned at birth?”. Sexuality was captured using self-identified categories (Heterosexual, Homosexual, Bisexual or Other). Religion was captured based on the most common categories in the original dataset (Christian, Hindu, Muslim, Other or no religion). To identify students with additional needs, the responses to whether students had any physical, mental or learning difficulties that meant they required additional support were used (Yes, No, Unsure). The level of study was captured using UK-based categories of education (e.g., Undergraduate is Level 4–6). First language was captured based on responses to the item “Is English your first language?” and birth country or immigrant status was captured based on responses to the item “Are you currently living in your birth country?”. Based on the available data and guidance from student consultations, students were categorised as marginalised based on the following criteria;

- Ethnicity–students who self-identified as belonging to a non-White ethnic group (e.g., Black, Asian, Mixed or Other)
- Sex/Gender expression–students who responded “no” to if they identify with the same sex assigned at birth and/or preferences for the pronouns “they/them” or “other”
- Sexuality—students who self-identified as belonging to a non- Heterosexual group (e.g., Homosexual, Bisexual or Other)
- Religious beliefs–students who identified with religious/spiritual beliefs that represented less than 50% of the general population (e.g., Hindu, Muslim, Other)
- Special education needs/disabilities–students who indicated they required additional support during their programme because of physical, mental or learning difficulties
- First language–students who responded “no” to “Is English your first language?”
- Country of birth–student who responded “no” to “Are you currently living in your birth country?”
- Age–mature students who were over 21 years of age at the beginning of their undergraduate studies or over 25 years of age at the beginning of their postgraduate studies [52]
- Intersectionality was defined as having multiple marginalised characteristics or belonging to more than one of the above categories [53].

#### BriefCOPE

Coping strategies/practices were assessed using the responses on the BriefCOPE, which is an abbreviated version of the Coping Orientation to Problems Experienced Inventory [54]. The BriefCOPE consisted of 28 items, and each item was rated on a 4-point Likert scale ranging from “I have not been doing this at all (score 1)” to “I have been doing this a lot (score 4)”. The scores corresponded to 14 dimensions, each reflecting the use of a coping strategy: active coping, planning, acceptance, denial, self-distraction, use of substance, use of emotional support, use of instrumental support, behavioural disengagement, venting, positive reframing, humour, religion, and self-blame. The BriefCope is a validated instrument with Cronbach’s alpha scores ranging from 0.50–0.90 [55]. We adopted the tri-categorisation for this scale that corresponded to problem-focused, emotion-focused, or avoidant coping styles [56].

#### Open-ended questions

The data from two open-ended questions were used to obtain students’ descriptions of their individual coping styles and mental health support needs. These items were developed based on consultations with students as described in the original study [46]. Therefore, responses to the following questions were included;

1. When you need additional support to improve your mental health and wellbeing, what do you do?
2. What role, if any, should your place of education (e.g., college or university) play in supporting your mental health and wellbeing?

### Data analysis

#### Quantitative data analysis

Continuous variables were summarised using means and 95% confidence intervals and presented based on the number of marginal characteristics and the corresponding category of coping styles. Categorical variables were summarised using frequencies. Linear regression modelling was applied to each of the three categories of coping strategies/practices (i.e., problem-focused, emotion-focused and avoidant) to assess the difference in coping strategy scores based on the number of marginalised characteristics. Beta coefficients, their standard errors and p-values were calculated. A two-tailed p-value of <0.05 was considered statistically significant. Quantitative data were analysed using the R Software, version 4.3.1 and SPSS, version 29.

#### Qualitative data analysis

The qualitative data were analysed using qualitative content analysis, which comprised of de-contextualization and re-contextualization of emerging concepts [57, 58]. More specifically, we were guided by the following steps: selecting the unit of analysis, coding all the data, revising the coding rules (if necessary), creating and defining the categories, revising the category scheme, and constructing themes [59]. First, familiarisation with the data was done through re-reading the quotes. Second, three researchers (SL, KB and JE) met to generate the initial coding of the data. Third, the team met weekly for recoding and clustering of the codes into potential themes. Based on consensus within the team, themes were considered dominant if there were more than n = 15 example quotes within the dataset. Qualitative data analysis and data management were conducted using Microsoft Excel and NVivo.

### Reflexivity, reliability, and rigour

During the study process, various strategies were adopted to maintain rigour and trustworthiness. The research team consisted of a diverse group of researchers with expertise in qualitative, quantitative and mixed-methods research, as well as extensive knowledge and practice in mental health and social work. Students with a particular interest in counselling and mental health programmes who were part of a research internship were included as co-researchers. Analysing both quantitative and qualitative data helped obtain different but complementary information that facilitated a deeper understanding of the mental health coping strategies and needs of marginalised students in the UK. As for the quantitative analysis, we used the BriefCOPE, which was found to be reliable and valid in previous studies [60, 61]. The team also included a statistician (MM) to guide the analysis and interpretation of the quantitative data. As for the qualitative study, we collaborated with students (JE, KB, SJ, NAR) to facilitate member checking and triangulation. Agreements prior to the data analysis were achieved through debriefing and weekly meetings. We used reflexivity among the qualitative data analysis team (SL, KB, JE, MO) for transparency and consistency. We discussed our views, experiences, and positionality on the topic. Shared notes, double coding and checking for inter-rater coding accuracy were incorporated as part of the audit trail and therefore were used for communicating and discussing the various perspectives towards data interpretation. Intersectionality and integration were captured in the interpretation and reporting of the findings. We also provided self-identified labels associated with direct quotes to support the transferability of our findings.

### Ethical considerations

The original study from which our dataset was identified was reviewed and ethically approved by the Research Ethics Committee of the Faculty of Health, Social Care and Medicine at Edge Hill University (ETH2021-0231). As this was an analysis of a subset of the anonymised data, and the participants provided consent for their data to be used for health and care research, no further ethical approvals were required [62].

## Results

### Characteristics of the sample

Data from 788 UK-based further and higher education students were included in our analysis. The average age of the total sample was 25 years (SD = 8.19, Range 16–77 years). Most students studied at the undergraduate level (485 or 61.5%) and were enrolled in full-time education (631 or 80.1%). Of the 788 students, 581 (73.7%) belonged to at least one marginalised group based on sex/gender (45 or 5.71%), language (103 or 13.07%), ethnicity (196 or 24.87%), birth country (156 or 19.8%), religious beliefs (74 or 9.39%), sexuality (193 or 24.49%), age (i.e., mature student status, 187 or 23.73%), and special education needs/disabilities (226 or 28.68%). 45.27% (263 out of 581) of the sample had multiple marginalised characteristics (Table 1).

**Table 1:** Number of students in each marginalised category.

N = 581	Age	Sex	Language	Ethnicity	Birth Country	Religion	Sexuality	SEN/D*
Age	187	12	20	33	34	13	38	65
Sex/Gender		45	5	3	7	0	39	27
First Language			103	39	90	22	23	18
Ethnicity				196	72	59	34	32
Birth Country					156	29	35	29
Religion						74	4	15
Sexuality							193	97
SEN/D*								226

*SEN/D = Special Education Needs and/or Disabilities

### Quantitative findings

#### Prevalence of coping strategies/practices

**Fig 2:**
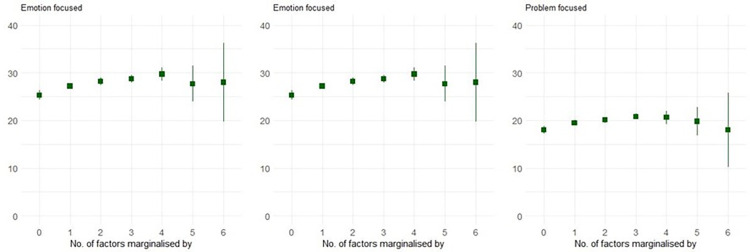
Means (95% CIs) based on marginalised characteristics.

Overall, significantly higher mean scores were observed for emotion-focused coping, followed by problem-focused and avoidant coping strategies (Table 2, Fig 2). Compared to students who were not categorised as marginalised (reference point 0), students who were marginalised based on one or more characteristic had significantly higher scores for problem-focused coping (M = 18(CI = 17.2,18.8) vs M = 20.1(CI = 19.5,20.7)), emotion-focused coping (M = 25.3(CI = 24.3,26.3) vs M = 28.2(CI = 27.4,29)) and avoidant coping (M = 14.7(CI = 14.1,15.3) vs M = 16.1(CI = 15.5,16.7)).

**Table 2:** Linear regression output for three categories of coping strategies.

		Avoidant focused		Emotion focused		Problem focused	
Number of characteristics marginalised by	# of students	Beta (SE)	P-value	Beta (SE)	P-value	Beta (SE)	P-value
Intercept		14.69(0.32)	0.000	25.31(0.44)	0.000	17.95(0.36)	0.000
0	207	Ref		Ref		Ref	
1	318	0.89(0.42)	0.032	1.89(0.56)	0.001	1.52(0.46)	0.001
2	333	1.42(0.41)	0.001	2.93(0.55)	0.000	2.15(0.46)	0.000
3	223	1.62(0.45)	0.000	3.43(0.6)	0.000	2.87(0.5)	0.000
4	57	2.13(0.7)	0.002	4.39(0.94)	0.000	2.68(0.77)	0.001
5	19	0.15(1.12)	0.892	2.42(1.5)	0.107	1.84(1.24)	0.138
6	3	4.64(2.71)	0.087	2.69(3.64)	0.461	0.05(3.01)	0.987

Note: Students who are not marginalised are considered as the reference category. The increasing number of marginalised characteristics represents intersectionality.

### Qualitative findings

#### Types of coping strategies/practices

Of the 581 students who were categorised as marginalised, 351 (60.41%) responses provided sufficient data (i.e., more than one-word responses) for the qualitative content analysis. To understand how students from marginalised groups cope with stressful life events, the excerpts were analysed and organised into six subthemes that aligned with the three overarching domains of emotion-focused, problem-focused, and avoidant coping. Coping strategies/practices mainly involved talking to friends and family, practising religion or spirituality, engaging in creative/innovative activities, using entertainment as a distraction, waiting to see if things improve and isolating. (n) represents the number of quotes that were identified in the dataset to support each subtheme.

#### Emotion-focused coping

Students described accessing social support to vent and seek comfort and advice. Although a smaller number of students used religious or spiritual practices like meditation or prayer to cope with stress (n = 20 example quotes present in the dataset), students most often referred to seeking support from friends and family members (n = 222).

*Talking to friends and family*. The primary source of support appeared to be romantic partners, close family members or friends, and fellow students. Students often spoke of meeting up with friends or family in person, but several students mentioned talking on the phone to express themselves and ‘get things off their minds’. For example, two students expressed;

“*My friends are my first port of call and after that my family*.*” (White bisexual female with special education needs/disabilities)*

“*My partner is a source of support as are my parents*…*” (Mixed-race male mature student)*

*Practising religion or spirituality*. A small number of students relied on religious or spiritual practices to cope with stress. Common practices included prayers, meditation, and yoga.

“…*My religion often helps improve my mental health*. *I often find myself turning to my religion (Islam)*. *It’s very comforting*…*” (Mixed-race Muslim female mature student)*

“*I like to participate in yoga to provide calm and clarity*.*” (Asian Hindu male)*

“…*I practice a lot of mindfulness*, *such as breathing and meditating which helps me to cope in the moment*.*” (White female with special education needs/disabilities)*

“*I meditate*…*” (Black female who immigrated to the UK)*

#### Problem-focused coping

Students described actively coping with stressors using different techniques. A small number of students reported taking prescribed medications (n = 13) to help them cope, and a few others also described taking time to think things through (n = 14) or using positive reframing to ‘*look at the bright side’* (n = 3). However, the most common strategy/practice was to engage in creative/innovative activities (n = 61) as a form of actively coping with stress.

*Engaging in creative/innovative activities*. Several students reported using creative and innovative activities such as technology-based tools like social media platforms or spending time outdoors to cope with stressors. Among these responses, it was common for students to report using diverse combinations of creative activities. For example;

“*I usually rely on well-established coping mechanisms such as exercising*, *playing my guitar and journaling*…*” (White female immigrant with special education needs/disabilities)*

“…*I focus on my hobbies like drawing and painting to wind down*…*” (White bisexual male immigrant)*

“…*I will go out for a walk in the woods to help ground me*…*I use an app*… *that helps you with self-care techniques and that helps prevent me from feeling terrible and stressed*.*” (White female bisexual mature student with special education needs/disabilities)*

#### Avoidant coping

Some students described avoiding dealing with stressors altogether by using distractions to help them disengage. This approach sometimes included unhelpful strategies like self-harming (n = 1) and substance use (n = 4). However, the more popular strategies/practices were using entertainment as a distraction (n = 28), waiting to see if things improved on their own (n = 24) and isolating (n = 19).

*Using entertainment as a distraction*. Some students chose to watch television, play computer games, read or listen to podcasts as a way of distracting themselves from life’s challenges. Other students also discussed how distractions in the form of entertainment temporarily helped them ignore what was happening around them. For the most part, students who described using entertainment as a distraction mentioned they chose this method as they found it difficult to talk to others about their problems.

“*I will listen to an audiobook or podcast that makes me feel better or play meditative music on YouTube*. *I find it hard to talk to therapists*. *I don’t know where to begin*.*” (Black female immigrant)*

“*Nothing but YouTube makes me happier; it’s a good distraction” (Student who identified as They/Them*, *Genderless*, *Unsure about their sexuality and had special education needs/disabilities)*

*Waiting to see if things improve*. Many students expressed that they mostly avoided confronting their problems by ‘waiting things out’ by sleeping in the hope that things improve by themselves. Again, some students disclosed this approach was easier as they struggled to reach out to others. For example;

“I *don’t do anything about it*, *I believe sleep just helps me pull through*…*” (Black Muslim male)*

“*I don’t do anything; just wait and see*.*” (Mixed-race Female immigrant mature student)*

“*I don’t really do anything*. *I just wait for it to pass*.*” (Female with special education needs/disabilities)*

*Isolating*. A number of students described self-isolation as a form of avoidance of facing the reality of their life. Students generally expressed that they kept things to themselves and avoided reaching out to others for support. Two students narrated;

“*I tend to keep things bottled up*…*”* (*White male mature student with special education needs/disabilities*)

“*I do not turn to anyone else [because] I’ve got trust issues*…*”* (*Black bisexual immigrant*)

In contrast, other students found self-isolation helpful to provide an element of self-care and self-preservation in order to ‘refuel’.

“…*I’m an introvert and I find time alone helps me recharge a bit” (White bisexual mature student)*

“*I prefer to just rest by myself rather than being around others” (Asian bisexual female with special education needs/disabilities)*

#### Support needs

To identify the mental health support needs among students from marginalised groups, the excerpts were analysed and organised into six subthemes that align with three overarching themes that centred around the need for improved or tailored well-being services, appropriate social support, and additional academic support. Support needs mainly involved increased awareness of mental health services, use of contemporary approaches to support mental health, frequent mentor/personal tutor support, opportunities for organised peer support, lowered academic pressures and reasonable adjustments.

#### Improved/Tailored mental health services

Although several students generally acknowledged that well-being services were available at their colleges and universities, students wanted some improvements which centred around increased awareness of the available support (n = 94) and more novel and contemporary approaches for accessing help (n = 69).

Increased awareness of mental health support

Students generally expressed a need for mental health support services to be more integrated into other parts of the academic process to increase opportunities for students to understand more about mental health and the available mental health support. Even when some students were aware of the services, such as counselling, they were not aware of alternatives that were suitable for different levels of stress or mental health problems. The following statements reflect the views of two students;

“*I had no idea how or who to contact at uni until recently and they don’t really make it clear what they can offer you*…*” (White pansexual transgendered student who identified as they/them)*

“…*No one speaks about it*.*” (White bisexual female mature student)*

To increase awareness, students made recommendations like frequent announcements to their email addresses, letters, or seminars. As illustrated by the following responses:

“*Carry out seminars and webinars to create awareness on mental health and wellbeing” (Black male mature student)*

“…*universities should send emails*, *letters or videos about how to improve mental health and let students know that they are there to support us all the time*.*” (Muslim student with special education needs/disabilities)*

*Use of contemporary approaches to support mental health*. Our analysis also highlighted a desire for alternative approaches to mental health support that appear to be more contemporary or different to traditional services. Students also described wanting more understanding and consideration from staff about their circumstances. This sometimes meant that students wanted tailored services to support minority groups. For example;

“*They [services] should be more understanding of students’ struggle because I am bisexual” (White bisexual female mature student)*

“*The support has been excellent but is perhaps tailored more to younger students” (White female mature student with special education needs/disabilities)*

Once students highlighted what could be improved, they proceeded to suggest various ways that services could support mental health. This included wanting increased accessibility to services and increased variety in what is delivered and how support is offered.

“*The only issue is the waiting list and limited number of sessions they can provide*, *can’t this be more flexible?” (White pansexual female)*

“*Anonymous online support systems that are free will be nice” (White bisexual female with special education needs/disabilities)*

#### Appropriate social support

Students generally appreciated the therapeutic support received from professionals at well-being services in educational settings. However, due to long waiting times and the fact students sometimes felt their problems were not severe enough to receive counselling, students expressed a need for other forms of social support like regular mentor/personal tutor support (= 39) and more opportunities for organised peer support (n = 17).

*Regular mentor/personal tutor support*. In terms of social support, students felt like they would benefit from opportunities for regular support sessions with personal tutors/mentors to enable them to form a positive rapport, subsequently encouraging discussions around mental health. One student expressed;

“*There should be semester check-ups with personal tutors or similar for every student” (White bisexual female)*

*Opportunities for organised peer support*. Some students also discussed how creating a community environment within the university, such as organised social-emotional groups, may help encourage therapeutic conversations with fellow peer groups. Students expressed a need for more opportunities to share experiences with like-minded peers in the hope that it may improve their wellbeing.

“*I wish that there were more chances to openly discuss mental health with other students like me*, *more group-therapy type of environments*…*” (Immigrant student with special education needs/disabilities)*

#### Additional academic support

Students expressed that their academic work kept them busy and sometimes acted as a distraction from other stressors in their lives. However, students expressed that academic pressures like strict deadlines sometimes led to mental health problems. This was further compounded by the inability of some tutors to show empathy during stressful periods. Therefore, to promote mental health and wellbeing, students wanted institutions to lower academic pressures placed on students (n = 39) and provide reasonable academic adjustments when needed (n = 32).

*Lowered academic pressures*. Since students expressed that academic pressure can be a source of stress, students suggested that lessening the pressures placed on assignments and shifting focus on wider learning would help to alleviate the stress. Thus. students wanted more empathy. This was summed up in one student’s response;

“*I think the process of gaining education has such a big impact on my levels of stress and wellbeing*. *If they [place of study] place stronger effort on students’ mental health and enjoyment*, *then this could lower the pressures*.*” (White bisexual male with special education needs/disabilities)*

*Reasonable academic adjustments*. In addition to lowering academic pressures, students wanted reasonable adjustments offered by tutors to ease stress. The most common adjustment mentioned was extensions on assignments. Other adjustments included online streaming of classes and flexibility with deadlines and learning. The following quotes illustrated this;

“*They should offer better measures like leeway with deadlines sometimes if you’re stressed*.*” (White immigrant female)*

“*Education should not worsen mental health situations*. *Education providers should try to find reasonable adjustments that can be made to assist*, *for example*, *allowing me to stream lectures from home*…*” (Asexual transgender student with special education needs/disabilities)*

### Integration of findings

**Fig 3:**
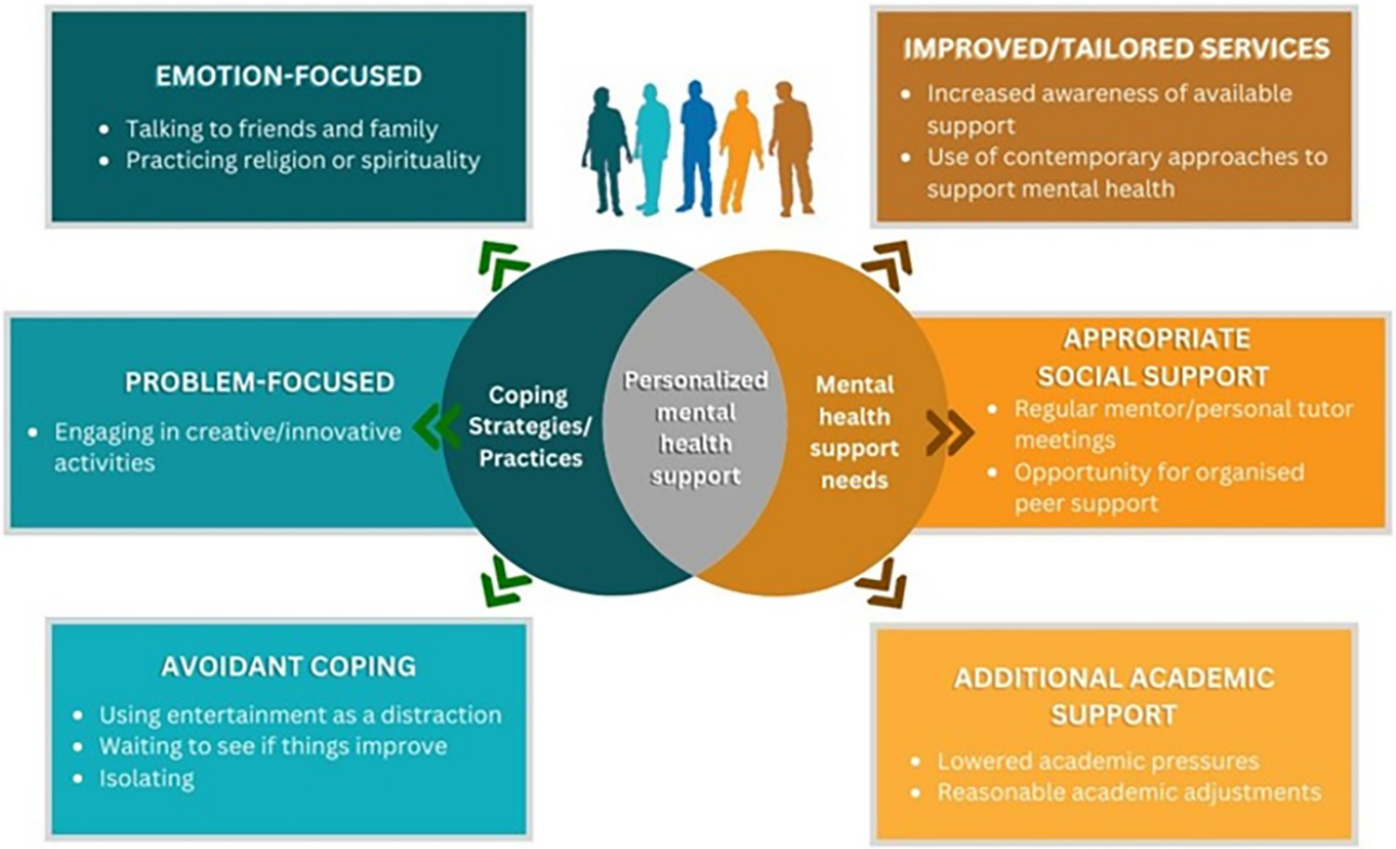
Conceptual framework of key findings.

Overall, students who were categorised as marginalised utilised a range of coping strategies to support their mental health and wellbeing. The overlap of social categories (i.e., intersectionality) appeared to result in increasing efforts to cope with life stressors. The popular coping strategies practised generally appeared to be harmless. For example, students viewed talking to friends and family members as a safe space to share worries and obtain support. However, there was a need for increased awareness of mental health services and the use of contemporary approaches to support mental health. At the integration level, the findings highlighted coping strategies and support needs that suggest the need to offer personalised, culturally appropriate early interventions to students at risk of experiencing mental health challenges (Fig 3).

## Discussion

### Summary of findings

The aims of this study were two-fold. First, to assess and understand the types and prevalence of different coping strategies/practices adopted by marginalised students. From the UK-based sample of 788 students, we observed that students who were marginalised based on one or more factors (n = 581) had significantly higher scores for problem-focused, emotion-focused and avoidant coping compared to other students (n = 207). Coping strategies/practices included talking to friends and family, practising religion or spirituality, engaging in creative/innovative activities, using entertainment as a distraction, waiting to see if things improve and isolating.

The second objective was to explore the mental health support needs of marginalised students. Based on our analysis of 351 cases, students mainly expressed a need for improved or tailored services, additional academic support, and appropriate social support. These included increased awareness of available mental health support, contemporary approaches to support mental health, regular mentor/personal tutor meetings, lowered academic pressures, reasonable academic adjustments when necessary and opportunities for organised peer support.

To the best of our knowledge, this is the only study to analyse a large sample (N = 581) of further and higher education students from multiple marginalised groups in the UK. Compared to previous research [63–66], our study is also novel as it pooled the data from multiple groups with multiple characteristics, which helped explore understudied areas like intersectionality. This study also analysed data from marginalised groups such as mature students, immigrants, and specific religious groups where there is a lack of mental health research among college and university students [67, 68].

### Interpretation of our findings

Our findings align with the existing evidence suggesting that students from marginalised groups adopt a wealth of mental health coping strategies, such as, talking to friends and family and religious or spiritual practices to promote acceptance, belonging and feelings of safety and security [69–71]. It is possible that the unique experiences of students from marginalised groups [23] resulted in a need for increased coping mechanisms, as highlighted in the current study. This may also explain the desire for appropriate social support. For example, peer-to-peer support, such as talking to other students and sharing experiences, supports socialising, which is fundamental in promoting wellbeing among students [72]. However, Gladstone et al [73] highlighted that marginalised groups may have reduced opportunities to develop these positive peer relationships due to negative experiences such as bullying or social exclusion within education settings [73]. As a result, it becomes important to provide such opportunities as part of an educational provision that is culturally aware of the needs of marginalised groups.

Our findings are also in line with previous research suggesting that religion and prayer can be prevalent coping strategies among marginalised groups and may act as a stress buffer while offering support, security and safety to students who may not have access to other services [74]. It is also well established that due to some cultural beliefs, some students may also seek support from religious leaders before accessing mental health services [75]. Previous research also concluded that stigma may still be one of the primary reasons some students may not seek support from others [76]. Therefore, this may further explain alternative coping strategies like isolation or waiting to see if things improve, which our study highlighted. Notably, our findings suggested that students may engage in creative or innovative activities as a form of self-help and use entertainment as a source of distraction to help cope with stress. Our findings build on previous research highlighting creativity and innovative activities such as hobbies and social media as useful strategies to support mental health and wellbeing [77, 79]. Yet creative and innovative activities with important therapeutic potential remain largely untapped within an educational environment. There is growing evidence suggesting that social media can be a protective factor against loneliness and impaired mental health [77]. Social media and online gaming are widely used among the student population, and therefore, this can also be a way to connect with people of similar interests, which may contribute to feelings of acceptance and belonging [78]. Other forms of creativity, like engaging with music and art, have also shown strong evidence for supporting mental health and wellbeing [79–82]. Thus, in the absence of social or professional connections, students from marginalised groups may use these strategies as a source of support. This also highlights further opportunities to incorporate creativity and physical activity among groups of students to encourage socialising and promote self-care and a sense of belongingness. These alternatives to verbal approaches could also help overcome language and cultural barriers that some students experience [83].

Our findings also add to the calls for increased awareness of mental health and mental health support among marginalised groups [84, 85]. Prior studies have also noted that increased awareness of mental health services may have a positive impact on mental health and wellbeing [27, 86, 87]. This, alongside difficulties in accessing appropriate services, can make it difficult for some students to seek professional help [88]. Therefore, more information about the types of services available and how to access them seems to be a recommendation for improved service provision among marginalised students in our sample. Some students highlighted that speaking to a counsellor was the only service they were aware of, and therefore, they did not always see their problems as requiring that level of support. This is an important finding suggesting that existing services may need to adopt new ways of sharing information about all services in addition to using diverse ways of delivery, such as the use of technology [89, 90]. This may also coincide with calls for more appropriate services, which are offered through online mediums to meet the preferences of some students. Similarly, students may benefit from alternative support from other professionals through mentorship and coaching [91]. It appears that students may not always have access or knowledge of this range of support [92]. To ensure appropriate services are offered, there have been several policy and advocacy calls to co-design support to ensure services are adapted to suit the needs of marginalised groups [89, 93]. This understanding is the foundation for developing and implementing more targeted and localised support mechanisms and interventions.

Our findings further suggest that regular mentor/personal tutor meetings may be a welcomed approach for supporting students’ mental health. Watts [92] highlighted that the personal tutor plays a pivotal role in shaping the student’s university experience, representing a focal point in the student’s interaction with the institution [94]. A study by Yale (2019) found that establishing an authentic and positive connection with one’s personal tutor was discovered to act as a protective factor, mitigating certain challenges faced during the first year of university and fostering a stronger sense of belonging [95].

Another expressed need was lowered academic pressures. In line with theories by Maslow (1943) and Rogers (1992), students may need psychological safety prior to focusing on and achieving academic success [40, 41]. Therefore, considerations for lowering academic pressures by introducing measures such as academic adjustments (e.g., assignment extensions, further assignment support, alternative assessment options) may be welcomed by students. This finding highlights the importance of creating a supportive environment so marginalised students can thrive academically and personally.

### Implications for practice, policy and future research

The voices of marginalised students represented in this study indicated the need for enhanced individualised services and academic and social support to better cope with stress and mental health issues. Undeniably, this may require student support services to increase their provision to offer more tailored services so all students can benefit [38, 39]. Therefore, to address this growing gap, more efficient strategies and efforts are also needed to implement sustainable approaches. Watkins et al (2021) propose a stepped care model where the intervention starts with an intensive, least resource, universal or whole university approach followed by targeted interventions [96, 97]. This model resonates with our study since marginalised students reported higher problem-focused coping strategies than other students. This could imply that these students are already utilising facets of active coping such as the use of informational support, planning, and positive reframing. Henceforth, promoting these inherent strengths, self-initiated care, and responsibility from students could facilitate a positive shift in the university culture from the ground up. To achieve this, education providers can aim to facilitate an environment for a compassionate and mindful education culture and nurture green spaces for engaging in creative, social and physical activities and other forms of self-care [4]. To tackle health inequities and offer support to students with severe difficulties, improved pathways to wellbeing services and tailored counselling and therapies are also necessary. This might include strategies designed to expand academic and social support initiatives and measures to address the needs highlighted by the participants of this study. However, it is not yet clear what approaches work best for whom as models of care vary across institutions and they have not been methodically evaluated rigorously [98]. Further research is needed to test the effective ways of offering universal and targeted care delivery to students and identify accessible pathways. Therefore, ongoing research may also be needed to co-design and evaluate new services and interventions as they are implemented.

Our findings also have implications for existing and revised guidance related to student wellbeing [99–101]. Whilst there is a growing body of advice provided for schools and colleges, the statutory and policy elements for higher education settings are still limited. The findings in this study highlight the importance of understanding the diversity of mental health needs and the importance of valuing insights regarding solutions to inform policy development. The argument for these changes can easily be made with respect to efforts to improve diversity and address social injustice amongst college and university student support services. Indeed, without strategies to widen the inclusivity of these services, there is a risk that they will, somewhat ironically, continue to exacerbate the challenges experienced by a large number of students.

### Limitations

Despite the strengths of this study, some limitations should be acknowledged. As this was a subsample of a larger dataset, the authors relied on the scope, quality and validity of the data from the original study [46]. Hence, since the primary study was conducted online, our findings may have a bias towards participants who have access to digital devices. Therefore the responses may not be representative of all marginalised students (e.g., low socioeconomic groups). We also acknowledge that mental health problems could have been heightened during the period of data collection based on the Covid-19 pandemic, so our findings may not be fully representative of the average daily stressors. Furthermore, within the qualitative data, the depth of information was limited as students replied to open-ended questions online, which meant some responses were brief. Further in-depth interviews can explore some of the themes raised in this study across marginalised groups and/or offer further information and understanding of specific subgroups. Although we pooled data from multiple groups with various characteristics, we acknowledge that individuals may attribute different meanings to each coping strategy and expressed need. Similarly, by exploring the mental health coping strategies and needs of specific subgroups, some individualised interventions could cause further discrimination. Therefore, it is important that codesign principles are adopted during intervention development, evaluation and implementation. Another limitation of this study is that we relied on self-report characteristics which meant it may not have been possible for some students to accurately identify as having SEN/D.

### Conclusions

This study described, for the first time, the mental health support needs and mental health coping strategies/practices of a large number of marginalised further and higher education students in the UK, bringing together the voices of diverse groups with diverse needs. We found that students who were marginalised based on one or more characteristics had significantly higher scores for problem-focused, emotion-focused and avoidant coping than other students. We also found that students needed improved or tailored services, additional academic support, and appropriate social support. These findings provide a unique and important knowledge base to inform policies, practice and future research to support typically underserved and underrepresented students in the UK.
